# Antimicrobial activities of saponins from *Melanthera elliptica* and their synergistic effects with antibiotics against pathogenic phenotypes

**DOI:** 10.1186/s13065-018-0466-6

**Published:** 2018-09-20

**Authors:** Cyrille Ngoufack Tagousop, Jean-de-Dieu Tamokou, Irene Chinda Kengne, David Ngnokam, Laurence Voutquenne-Nazabadioko

**Affiliations:** 1 Research Unit of Environmental and Applied Chemistry, Department of Chemistry, Faculty of Science, University of Dschang, P.O. Box 67, Dschang, Cameroon; 2 Research Unit of Microbiology and Antimicrobial Substances, Department of Biochemistry, Faculty of Science, University of Dschang, P.O. Box 67, Dschang, Cameroon; 3 Groupe Isolement et Structure, Institut de Chimie Moléculaire de Reims (ICMR), CNRS, UMR 7312, Bat. 18, BP.1039, 51687 Reims cedex 2, France

**Keywords:** *Melanthera elliptica*, Asteraceae, Saponins, Antibacterial, Antifungal, Synergy, Additive

## Abstract

**Background:**

Resistance of bacteria and fungi to antibiotics is one of the biggest problems that faces public health. The present work was designated to evaluate the antimicrobial activities of saponins from *Melanthera elliptica* and their synergistic effects with standard antibiotics against pathogenic phenotypes. The plant extract was prepared by maceration in methanol. The methanol extract was partitioned into ethyl acetate and *n*-butanol extracts. Column chromatography of the *n*-butanol extract followed by purification of different fractions led to the isolation of four saponins. Their structures were elucidated on the basis of spectra analysis, and by comparison with those from the literature. The antimicrobial activities of the extracts/compounds alone and their combinations with tetracycline and fluconazole were evaluated using the broth microdilution method through the determination of minimum inhibitory concentration (MIC) and minimum microbicidal concentration.

**Results:**

Four compounds: 3-*O*-*β*-d-glucuronopyranosyl-oleanolic acid (**1**), 3-*O*-*β*-d-glucuronopyranosyloleanolic acid 28-*O*-*β*-d-glucopyranosyl ester (**2**), 3-*O*-*β*-d-glucopyranosyl(1 → 2)-*β*-d-glucuronopyranosyl oleanolic acid (**3**) and 3-*O*-*β*-d-glucopyranosyl(1 → 2)-*β*-d-glucuronopyranosyl oleanolic acid 28-*O*-*β*-d-glucopyranosyl ester (**4**) were isolated. Compounds **1**, **2** and **3** showed the largest antibacterial activities (MIC = 8–128 μg/mL) whereas compound **4** displayed the highest antifungal activities (MIC = 8–16 μg/mL). The antibacterial activities of compounds **1** and **2** (MIC = 16–32 μg/mL) against multi-drug-resistant *Escherichia coli* S2 (1) and *Shigella flexneri* SDINT are equal to those of vancomycin (MIC = 16–32 μg/mL) used as reference antibiotic.

**Conclusions:**

The present study showed significant antimicrobial activity of compounds **1**, **2**, **3** and **4** against the tested microorganisms. The saponins act in synergy with the tested standard antibiotics. This synergy could lead to new options for the treatment of infectious diseases and emerging drug resistance.

## Introduction

The development and spread of resistance to currently available antibiotics is a global concern [1]. Many investigations have reported multidrug-resistant pathogens, including *Escherichia coli*, *Pseudomonas aeruginosa*, *Shigella flexneri*, *Staphylococcus aureus*, *Candida*, *Cryptococcus*, *Aspergillus* and *Fusarium* [2, 3]. With the increase in bacterial and fungal resistance to antibiotics, plant-based antimicrobials have attracted attention in the scientific research. The use of natural antimicrobial compounds is important not only in food preservation, but also in the control of human and plant infectious diseases [4]. The use of natural products with therapeutic properties, whether vegetable, animal, and mineral, for a long time were the main sources of important therapeutic agents as well as important raw materials for the manufacture of traditional and modern medicines [5]. Many bioassay reports have indicated the presence of antimicrobial compounds including saponins from several medicinal plants in Cameroon [6–8]. The combination of saponins with antibiotics therapeutic approach may lead to new ways to treat infectious diseases. Therefore, many researchers have studied experimentally the synergistic effect resulting from the association of antibiotics with various plant extracts [9–12].

Saponins constitute a large group of glycosides having in their breasts a hydrophilic part consisting of monosaccharides and a lipophilic part commonly called genin. They are characterized by their surfactant properties because they dissolve in water forming foaming solutions. They are abundant in many plants and divided into two groups namely steroidal saponins and triterpenoid saponins. Many biological activities of saponins have been reported such as antibacterial, antifungal, antiviral, anti-inflammatory, anti-ulcer, haemolytic and hepatoprotective properties [13–18]. In addition, the saponins demonstrated the potential to act synergistically with antibiotics, contributing to the recycling of old antibiotics that were once considered ineffective due to resistance problems [19–22]. Hence, the synergistic effect of combinations of saponins with standard antibiotics will open a complete new field of possible applications in medicine to overcome resistance in multidrug resistant microorganisms.

*Melanthera elliptica* O. Hoffm., belonging to Asteraceae family, is a perennial bushy herb to 1.3 m high, commonly found in tropical West and Central Africa [23]. The leaves are used in folk medicine to treat cough, malaria and stomach ache [24]. In Cameroon, the herbalists use the leaf decoction to treat gonorrhea, feverish headache and pulmonary haemorrhage. The aqueous and ethanol whole plant extract of *Melanthera scandens* showed antibacterial activities against a group of microorganisms that are implicated in either typhoid fever and/or other gastrointestinal disorders such as diarrhea and dysentery [25, 26]. The phytochemical screening of the ethanolic extract of the leaves of *M. scandens* revealed the presence of cardiac glycosides, tannins, saponins, terpenes and flavonoids [27]. To the best of our knowledge no study has been carried on the phytochemical analysis and antimicrobial properties of *M. elliptica.* This work was carried out in order to evaluate the antimicrobial activities of saponins from *M. elliptica* and their synergistic effects with standard antibiotics against pathogenic phenotypes namely *E. coli*, *S. flexneri*, *S. aureus*, *Candida albicans*, *Candida parapsilosis* and *Cryptococcus neoformans*.

## Methods

### General experimental procedures

The isolation of secondary metabolites was done using column chromatography on Merck silica gel 60 (70–230 mesh) and gel permeation on Sephadex LH-20. TLC was carried out on silica gel GF254 pre-coated plates developed with ternary systems consisting of AcOEt–MeOH–H_2_O in the proportion (8-1-1 or 8-2-1). Detection was accomplished by spraying with 50% H_2_SO_4_ followed by heating at 100 °C, or by visual inspection under UV lamp at 254 and 365 nm. In order to determine the structures of the isolated compounds, the 1D and 2D NMR spectra were made. In fact, ^1^H and ^13^C NMR spectra were recorded on a Bruker Avance III 600 spectrometer equipped with a cryoprobe (^1^H at 600 MHz and ^13^C at 150 MHz). The 2D NMR experiments were recorded using Bruker microgram firmware (Xwin-NMR software version 2.1 TopSpin 3.2). Chemical shifts (*δ*) were reported in parts per million (ppm) using the residual solvent signals as a secondary reference to the TMS (*δ* = 0), while the coupling constants (*J* values) are given in Hertz (Hz).

### Plant material

The aerial parts of *M. elliptica* were collected at FOTO Village (Menoua Division, Western region of Cameroon) in January 2014. Authentication was done by Mr. Fulbert Tadjouteu, a Botanist of the Cameroon National Herbarium, Yaoundé, where a voucher specimen (N°8002/HNC) was deposited.

### Extraction and isolation

The air-dried plant material (4 kg) was powdered and extracted at room temperature with methanol (3 × 20 L, 72 h). The solvent was evaporated under reduced pressure, leaving an extract (220 g). Part of this extract (210 g) was suspended in water (300 mL) and successively extracted with equal volumes (500 mL) of ethyl acetate (EtOAc) and *n*-BuOH (saturated with water) yielding respectively 65 g and 30 g extracts after evaporation to dryness.

A part of the *n*-BuOH extract (25 g) was subjected to silica gel column chromatography using EtOAc–MeOH (100:0 → 40:60) gradient elution. Forty-seven fractions of 250 mL each were collected and combined on the basis of their TLC profiles to give five fractions: A (1–7), B (8–12), C (13–15), D (16–47) and E (24–47). Fraction B (3 g) was also purified on silica gel column chromatography eluted with EtOAc to give compound **1** (75 mg). Fraction C (5 g) mainly yielded compounds **2** (30 mg) and **3** (30 mg) after multiple chromatographic steps over silica gel using EtOAc–MeOH–H_2_O (95:5:2) and (90:5:5) as eluents. Fraction D (6 g) eluted by EtOAc–MeOH (80:20) on column chromatography separation over silica gel yielded three sub-fractions (D_1_–D_3_). Compounds **4** (35 mg) was obtained from fraction D_2_ after multiple chromatography separation over silica gel using EtOAc–MeOH–H_2_O (80:10:10) and (80:20:10) as eluents.

### Antimicrobial assay

#### Microorganisms

The studied microorganisms were three strains of bacteria (*S. aureus* ATCC 25923, *E. coli* S2 (1) and *S. flexneri* SDINT) and three strains of yeasts (*C. parapsilosis* ATCC 22019, *C. albicans* ATCC 9002 and *C. neoformans* IP95026) taken from our laboratory collection. The bacterial and fungal species were grown at 37 °C and maintained on nutrient agar (NA, Conda, Madrid, Spain) and Sabouraud Dextrose Agar (SDA, Conda) slants respectively.

#### Determination of minimum inhibitory concentration (MIC) and minimum microbicidal concentration (MMC)

Minimum inhibitory concentration values were determined by a broth micro-dilution method as described earlier [28] with slight modifications. Each test sample was dissolved in dimethylsulfoxide (DMSO) and the solution was then added to Mueller Hinton Broth (MHB) for bacteria or Sabouraud Dextrose Broth (SDB) for yeasts to give a final concentration of 8192 μg/mL. This was serially diluted twofold to obtain a concentration range of 0.125–4096 μg/mL. Then, 100 μL of each concentration were added in each well (96-well microplate) containing 95 μL of MHB or SDB and 5 μL of inoculum for final concentrations varying from 0.0625 to 2048 μg/mL. The inoculum was standardized at 2.5 × 10^5^ cells/mL for yeasts and 10^6^ CFU/mL for bacteria using a JENWAY 6105 UV/Vis spectrophotometer. The final concentration of DMSO in each well was <  1% [preliminary analyses with 1% (v/v) DMSO did not inhibit the growth of the test organisms]. The negative control well consisted of 195 μL of MHB or SDB and 5 μL of the standard inoculum. The cultured micro plates were covered; then, the contents of each well were mixed thoroughly using a plate shaker (Flow Laboratory, Germany) and incubated at 35 °C for 24 h (bacteria) and 48 h (yeasts) under shaking. The assay was repeated three times. The MIC values of samples were determined by adding 50 μL of a 0.2 mg/mL *p*-iodonitrotetrazolium violet solution followed by incubation at 35 °C for 30 min. Viable microorganisms reduced the yellow dye to a pink color. MIC values were defined as the lowest sample concentrations that prevented this change in color indicating a complete inhibition of microbial growth. For the determination of MMC values, a portion of liquid (5 μL) from each well that showed no growth of microorganism was plated on Mueller Hinton Agar or SDA and incubated at 35 °C for 24 h (for bacteria) or 35 °C for 48 h (for yeasts). The lowest concentrations that yielded no growth after this subculturing were taken as the MMC values [29]. Vancomycin (Sigma-Aldrich, Steinheim, Germany) and fluconazole (Merck, Darmstadt, Germany) were used as positive controls for bacteria and yeasts, respectively.

#### Combined effect of antibiotics and isolated compounds

The interaction between combination of reference antibiotics and isolated compounds (**1**, **3** and **4**) was performed by using the broth microdilution method. The antimicrobial activity of the isolated compounds in the presence of reference antibiotics (1/4 × MIC) and that of reference antibiotics in the presence of the isolated compounds (1/4 × MIC) were evaluated as described above. The preliminary tests allowed the selection of MIC/4 as the sub-inhibitory concentrations of the samples. The fractional inhibitory concentration (FIC) index for combinations of two antibacterial agents was calculated according to the following equation: FIC index = FIC A + FIC C; where FIC A = MIC of antibiotic in combination/MIC of antibiotic alone; FIC C = MIC of the isolated compound in combination/MIC of the isolated compound alone. The FIC indices were interpreted as follows: ≤ 0.5, synergy; > 0.5–1, addition; > 1 and ≤ 4, indifference and > 4, antagonism [30]. All the experiments were performed in triplicate.

## Results

### Chemical analysis

**Fig. 1:**
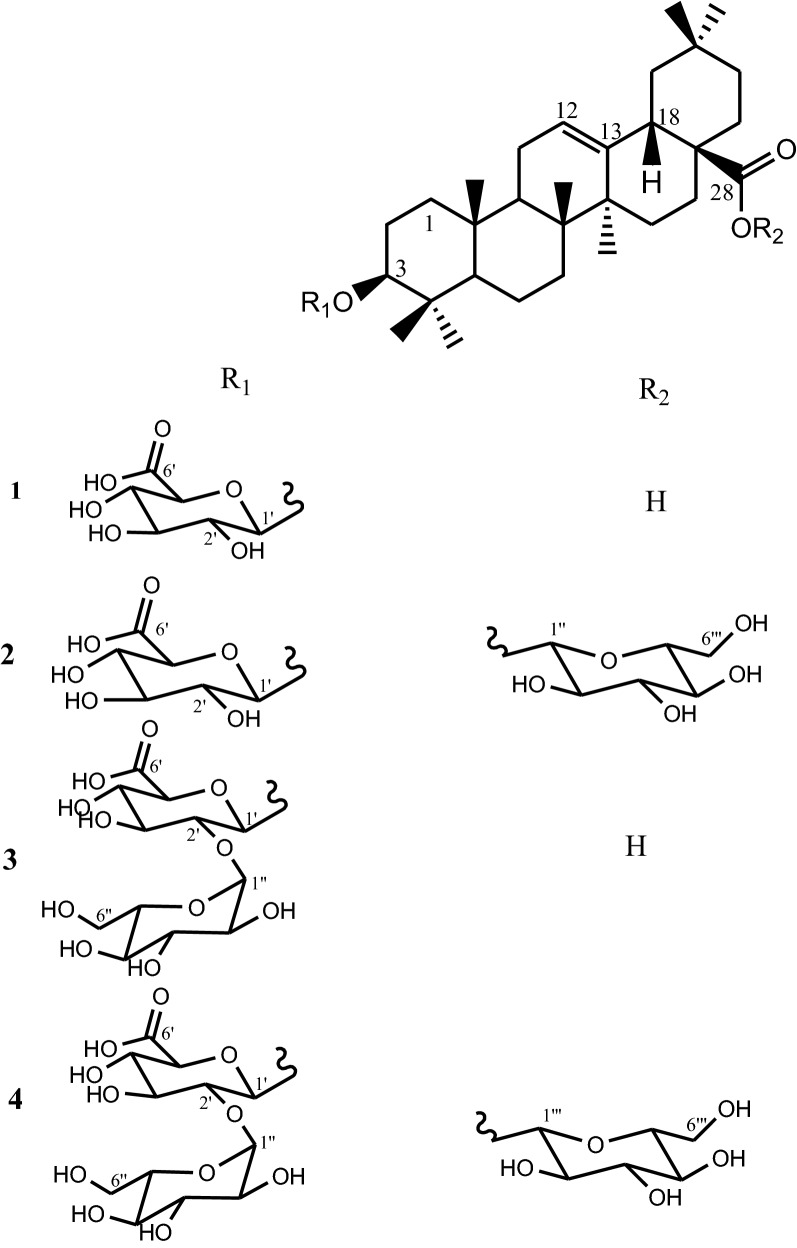
Chemical structures of saponins (**1**–**4**) isolated from *n*-BuOH extract of areal parts of *M. elliptica*. **1**: 3-*O*-*β*-d-glucuronopyranosyl-oleanolic acid; **2**: 3-*O*-*β*-d-glucuronopyranosyloleanolic acid 28-*O*-*β*-d-glucopyranosyl ester; **3**: 3-*O*-*β*-d-glucopyranosyl(1 → 2)-*β*-d-glucuronopyranosyl oleanolic acid; **4**: 3-*O*-*β*-d-glucopyranosyl(1 → 2)-*β*-d-glucuronopyranosyloleanolic acid 28-*O*-*β*-d-glucopyranosyl ester

The purification of *n*-BuOH extract of aerial parts of *M. elliptica* led to the isolation of four saponins (Fig. 1). Structures of these compounds were assigned on the basis of spectroscopic data (^1^H and ^13^C NMR, ^1^H-^1^H COSY, HSQC, HMBC, and ROESY) and by the comparison of their spectroscopic data with those reported in the literature. These compounds are: 3-*O*-*β*-d-glucuronopyranosyl-oleanolic acid (**1**) [31], 3-*O*-*β*-d-glucuronopyranosyloleanolic acid 28-*O*-*β*-d-glucopyranosyl ester (**2**) [31], 3-*O*-*β*-d-glucopyranosyl(1 → 2)-*β*-d-glucuronopyranosyl oleanolic acid (**3**) [32] and 3-*O*-*β*-d-glucopyranosyl(1 → 2)-*β*-d-glucuronopyranosyl oleanolic acid 28-*O*-*β*-d-glucopyranosyl ester (**4**) [33].

### Antimicrobial activity of the isolated saponins

The antimicrobial activities of MeOH, *n*-BuOH and EtOAc extracts as well as their isolated compounds were examined by microdilution susceptibility assay against three bacterial and three fungal strains selected on the basis of their relevance as human pathogens. The results showed that the extracts and isolated compounds exhibited variable MICs, depending on the microbial strains (Table 1). The MeOH and *n*-BuOH extracts were active only against bacterial species whereas the EtOAc extract displayed antimicrobial activity against both bacterial and fungal species. The EtOAc extract was the most active extract followed in decreasing order by *n*-BuOH and MeOH extracts.

**Table 1:** Antimicrobial activity (MIC and MMC in µg/mL) of extracts, isolated compounds and reference antimicrobial drugs

Extracts/compounds	Inhibition parameters	*E. coli*	*S. flexneri*	*S. aureus*	*C. parapsilosis*	*C. albicans*	*C. neoformans*
MeOH extract	MIC	512	256	128	> 2048	> 2048	> 2048
	MMC	1024	512	128	/	/	/
	MMC/MIC	2	2	1	/	/	/
*n*-BuOH extract	MIC	256	128	128	> 2048	> 2048	> 2048
	MMC	512	256	128	/	/	/
	MMC/MIC	2	2	1	/	/	/
EtOAc extract	MIC	128	128	64	1024	1024	512
	MMC	256	128	128	1024	1024	1024
	MMC/MIC	2	1	2	1	1	2
**1**	MIC	32	16	8	32	64	16
	MMC	32	32	16	64	64	16
	MMC/MIC	1	2	2	2	1	1
**2**	MIC	32	16	16	> 256	> 256	32
	MMC	32	32	16	/	/	64
	MMC/MIC	1	2	1	/	/	2
**3**	MIC	128	64	32	128	128	32
	MMC	256	128	64	256	256	64
	MMC/MIC	2	2	2	2	2	2
**4**	MIC	256	128	64	16	16	8
	MMC	> 256	256	128	32	64	16
	MMC/MIC	/	2	2	2	4	1
Ref^a^	MIC	32	16	0.5	0.5	1	2
	MMC	32	16	0.5	0.5	1	2
	MMC/MIC	1	1	1	1	1	1

/: not determined;* MIC* minimum inhibitory concentration,* MMC* minimum microbicidal concentration

^a^Fluconazole for yeasts and vancomycin for bacteria

The lowest MIC value was recorded on *S. aureus* with compound 1 and on *C. neoformans* with compound **4**, whereas the lowest MBC value was obtained on *S. aureus* with compounds **1** and **2** and on *C. neoformans* with compounds **1** and **4**. However, the highest MIC value was recorded on *C. parapsilosis* and *C. albicans* with compound **1** and the highest MBC value was obtained on *C. parapsilosis, C. albicans* and *C. neoformans* with compound **1**. The antibacterial activities of compounds **1** and **2** against *E. coli* S2 (1) and *S. flexneri* SDINT are equal to those of vancomycin used as reference antibiotic.

### Antimicrobial activity of the combination between antibiotics and saponins

The effect of the interaction between the isolated saponins (**1**, **3** and **4**) and antibiotics (vancomycin and fluconazole) was studied and the results are depicted in Tables 2, 3 and 4. The MIC values of the isolated saponins in combination with antibiotics at the concentration of MIC/4 are smaller than that of the isolated saponins used alone, suggesting an increase in the activity of saponins in combination with antibiotics (Table 2). The MIC values of antibiotics (vancomycin and fluconazole) in combination with the isolated saponins at the concentration of MIC/4 are also smaller than those of vancomycin or fluconazole alone, indicating an increase in the activity of vancomycin or fluconazole in combination with the isolated saponins at the concentration of MIC/4 (Table 3).

**Table 2:** Antimicrobial activities of the isolated compounds in the presence of reference antibiotics^a^ at 1/4 of MIC as a function of microorganisms

Microorganisms	Compound **1** alone	Compound **1** with ATB^a^ at 1/4 of MIC		Compound **3** alone	Compound **3** with ATB^a^ at 1/4 of MIC		Compound **4** alone	Compound **4** with ATB^a^ at 1/4 of MIC	
	MIC	MIC	FIC	MIC	MIC	FIC	MIC	MIC	FIC
*E. coli*	32	8	0.25	128	16	0.125	256	16	0.0625
*S. flexneri*	16	8	0.5	64	16	0.25	128	4	0.0312
*S. aureus*	8	1	0.125	32	16	0.50	64	2	0.0312
*C. parapsilosis*	32	4	0.125	128	4	0.0312	16	2	0.125
*C. albicans*	64	4	0.0625	128	4	0.0312	16	1	0.0625
*C. neoformans*	16	2	0.125	32	2	0.0625	8	0.5	0.0625

*MIC* minimal inhibitory concentration, *FIC* fractional inhibitory concentration, *ATB* antibiotics

^a^Fluconazole for yeasts and tetracycline for bacteria were tested at the concentration of MIC/4 on the corresponding microorganism

**Table 3:** Antimicrobial activities of the reference antibiotics in the presence of compounds 1, 3 and 4 at 1/4 of MIC as a function of microorganisms

Microorganisms	ATB^a^ alone	ATB^a^ with compound 1 at 1/4 of MIC		ATB^a^ with compound 3 at 1/4 of MIC		ATB^a^ with compound 4 at 1/4 of MIC	
	MIC	MIC	FIC	MIC	FIC	MIC	FIC
*E. coli* S2 (1)	32	1	0.0312	0.25	0.00781	0.125	0.00390
*S. flexneri*	16	0.25	0.0156	0.125	0.00781	0.0312	0.00195
*S. aureus*	2	0.125	0.0625	0.0625	0.0312	0.0312	0.0156
*C. parapsilosis*	0.5	0.25	0.50	0.0625	0.125	0.0312	0.0625
*C. albicans*	1	0.125	0.125	0.0625	0.0625	0.0625	0.0625
*C. neoformans*	2	0.125	0.0625	0.0625	0.0312	0.0312	0.0156

*MIC* minimal inhibitory concentration, *FIC* fractional inhibitory concentration, *ATB* antibiotics

^a^Fluconazole for yeasts and tetracycline for bacteria; compounds **1**, **3** and **4** were tested at the concentration of MIC/4 on the corresponding microorganism

**Table 4:** Fractional inhibitory concentrations (FIC) calculated for the combination of reference antibiotics and compounds 1, 3 and 4 as a function of microorganisms

Microorganisms	Compound **1 **+ ATB^a^		Compound **3 **+ ATB^a^		Compound **4 **+ ATB^a^	
	∑ FIC	Interpretation	∑ FIC	Interpretation	∑ FIC	Interpretation
*E. coli*	0.2812	Synergy	0.132	Synergy	0.0664	Synergy
*S. flexneri*	0.515	Additive	0.257	Synergy	0.0331	Synergy
*S. aureus*	0.25	Synergy	0.531	Additive	0.0468	Synergy
*C. parapsilosis*	0.625	Additive	0.156	Synergy	0.187	Synergy
*C. albicans*	0.187	Synergy	0.0937	Synergy	0.125	Synergy
*C. neoformans*	0.187	Synergy	0.0937	Synergy	0.0781	Synergy

*Σ FIC* sum of fractional inhibitory concentrations,* ATB* antibiotics

^a^Fluconazole for yeasts and tetracycline for bacteria

According to the obtained results, the combination of compound **1** with tetracycline showed a synergistic effect against *E. coli* and *S. aureus* while an additive effect was observed in *S. flexneri* (Table 4). The application of fluconazole with compound **1** led to a synergistic effect on *C. albicans* and *C. neoformans* and to an additive effect on *C. parapsilosis*. Combination of compound **3** with tetracycline had a synergistic effect against *E. coli* and *S. shigella*, and an additive effect against *S. aureus*. A synergistic effect was recorded on *C. parapsilosis*, C. *albicans* and *C. neoformans* with the combination of compound **3** and fluconazole. The interaction between compound **4** and tetracycline/fluconazole led to a synergistic effect against the tested bacterial and fungal strains. The combinatory effect of saponins and antibiotics was tested against six pathogenic microorganisms including two multi-drug-resistant (MDR) pathogenic bacteria [*E. coli* S2 (1) and *S. flexneri* SDINT]. The MIC of tetracycline changed to the susceptible range (MIC: ≤ 1 mg/mL) when saponins at the concentration of MIC/4 was added. This result provides strong evidence for the therapeutic potential of saponins in combination with defined antibiotic drugs to fight MDR pathogenic bacteria.

## Discussion

The findings of the present study indicated that the MeOH, *n*-BuOH and EtOAc extracts as well as their isolated saponins showed antimicrobial activity against different bacterial and fungal strains, but at different levels. According to the literature, saponins and extracts of *Melanthera scandens* are known for their antibacterial activities [15, 18, 25, 26]. The results of this study also revealed that all the tested microorganisms were sensitive to EtOAc extract, suggesting that the fractionation enhanced the antimicrobial activity of this extract and diluted that of *n*-BuOH extract. According to Tamokou et al. [34], a plant extract is considered to be highly active if the MIC < 100 μg/mL; significantly active when 100 ≤ MIC ≤ 512 μg/mL; moderately active when 512 < MIC ≤ 2048 μg/mL; weakly active if MIC > 2048 μg/mL and not active when MIC > 10 mg/mL. Hence, the *n*-BuOH and MeOH extracts were significantly active (100 ≤ MIC ≤ 512 μg/mL) against the bacterial strains and weakly active (MIC > 2048 μg/mL) against the tested yeasts whereas the EtOAc extract was highly active (MIC < 100 μg/mL) against *S. aureus*, significantly active on *E. coli*, *S. flexneri* and *C. neoformans* and moderately active (512 < MIC ≤ 2048 μg/mL) vis-à-vis *C. parapsilosis* and *C. albicans*. These findings highlight the traditional use of the *M. elliptica* in the treatment of infectious diseases, especially those caused by the tested microorganisms.

Antimicrobial cut-off points of pure compounds have been defined as follows: highly active: MIC below 1 µg/mL (or 2.5 µM), significantly active: 1 ≤ MIC ≤ 10 µg/mL (or 2.5 ≤ MIC < 25 µM), moderately active: 10 < MIC ≤ 100 µg/mL (or 25 < MIC ≤ 250 µM), weakly active: 100 < MIC ≤ 1000 µg/mL (or 250 < MIC ≤ 2500 µM and not active: MIC > 1000 µg/mL (or > 2500 µM) [34]. Based on this, the antimicrobial activity of compound **1** on *S. aureus* as well as that of compound **4** on *C. neoformans* can be considered significant. The strain of *E. coli* S2 (1) and *S. flexneri* [12, 35, 36] included in the present study were MDR clinical isolates and these were resistant to commonly used drugs such as tetracycline, streptomycin, ampicillin, nalidixic acid, *co*-trimoxazole, furazolidone, etc. However, most of the tested saponins displayed moderated antibacterial activities against these bacterial strains, suggesting that their administration may represent an alternative treatment against multidrug resistant *E. coli* and *S. flexneri*.

With regard to the structure–activity relationship analysis, the four saponins with the same basic skeleton, showed different degrees of antimicrobial activity. Compounds **1**, **2** and **3** with less than 3 sugar moieties showed the largest antibacterial activities with the best MIC value recorded with compound **1** on *S. aureus* whereas compound **4** with 3 sugar moieties displayed the highest antifungal activities with the best MIC value recorded with this compound on *C*. *neoformans*. In general, compound **1** was the most active followed in decreasing order by compound **4**, compound **3** and compound **2**. These observations show that the sugar moieties should be responsible for the difference in the observed activity.

The Gram-positive bacterium, *S. aureus* was found to be more sensitive compared to Gram-negative bacteria, *E. coli* and *S. flexneri*. The findings of the present study showed that the antimicrobial activities varied with the bacterial and fungal strains. These variations may be due to genetic differences between the microorganisms.

With regard to the MIC and MMC values, a lower MMC/MIC (≤ 4) value signifies that a minimum amount of plant extracts/pure compounds is used to kill the microbial species, whereas, a higher value signifies the use of comparatively more amount of sample for the control of any microorganism [37].

Combined antibiotic therapy has been shown to delay the emergency of microbial resistance and may also produce favorable synergistic effects in the treatment of infectious diseases. Drug synergism between known antibiotics and bioactive plant extracts is one of the novel ways to overcome the resistance mechanisms of microorganisms. Since lipophilic saponins targeting the biomembrane usually accompany polar polyphenols in phytomedical preparations, we decided to investigate their effect as single substances and in combination with standard antibiotics to gain further insight into potential synergistic effects of herbal medicine. In this study, the application of saponins with tetracycline or fluconazole showed synergistic effect on most of the tested microbial strains. This is very promising since the observed synergistic effect could lead to new options for the treatment of infectious diseases and emerging drug resistance. This result corroborates that of Brahim et al. [38] who showed that the combination of saponins extracts and fluconazole exhibited a total synergism against *C. albicans*, *C. parapsilosis*, *Candida krusei* and *Candida glabrata*. Synergistic activities were also verified when combining saponins with tetracycline, erythromycine and ciprofloxacine against *S. aureus* XU212, *S. aureus* RN4220 and *S. aureus* SA1199B [39]. The saponin of *Quillaja saponaria* enhanced the sensitivity of the Gram-negative bacteria *Proteus mirabilis* towards colistin [21]. In a murine wound model induced by *S. aureus* or *E. faecalis* infected BALB/c mice, the coapplication of local saponin (HSM-1) and intraperitoneal treatment with vancomycin or daptomycin resulted in a higher reduction of bacteria in infected BALB/c mice as compared to the respective antibiotic treatment alone [22]. These observations reinforce the advantageous antimicrobial effect of phytochemical-antibiotic combinations.

## Conclusions

The overall results of the present work provide baseline information for the possible use of *M. elliptica* extracts and mostly the isolated saponins in the treatment of infectious diseases caused by the tested microorganisms. In addition, the isolated saponins of this plant could be used in association with tetracycline and fluconazole to combat multidrug resistant pathogens subject to further toxicological and pre-clinical studies.

## Abbreviations

^13^C-NMR: carbon thirteen nuclear magnetic resonance
^1^H NMR: proton nuclear magnetic resonance
2D NMR: two-dimension nuclear magnetic resonance
ATCC: American Type Culture Collection
CC: column chromatography
COSY: correlation spectroscopy
DMSO: dimethylsulfoxide
EtOAc: ethyl acetate
HMBC: heteronuclear multiple bond connectivities
HNC: *Herbier National du Cameroun*
HR-EI-MS: high resolution electron impact mass spectrometry
HR-TOFESIMS: high-resolution time of flight electrospray ionization mass spectrometry
HSQC: the heteronuclear single quantum coherence
IR: infra-red
MBC: minimum bactericidal concentration
MDR: multi-drug-resistant
MeOH: methanol
MHA: Mueller–Hinton agar
MHB: Mueller–Hinton broth
MIC: minimum inhibitory concentration
MMC: minimum microbicidal concentration
NA: nutrient agar
*n*-BuOH: *n*-butanol
NMR: nuclear magnetic resonance
Rf: retention factor
TLC: thin layer chromatography
TMS: tetramethylsilane
TOF-ESIMS: time of flight electrospray ionization mass spectrometry
UV: ultra-violet
